# Eye movements during visual search in patients with glaucoma

**DOI:** 10.1186/1471-2415-12-45

**Published:** 2012-08-31

**Authors:** Nicholas D Smith, Fiona C Glen, David P Crabb

**Affiliations:** 1 Department of Optometry and Visual Science, City University London, London, UK

**Keywords:** Glaucoma, Eye movements, Visual search, Visual disability

## Abstract

**Background:**

Glaucoma has been shown to lead to disability in many daily tasks including visual search. This study aims to determine whether the saccadic eye movements of people with glaucoma differ from those of people with normal vision, and to investigate the association between eye movements and impaired visual search.

**Methods:**

Forty patients (mean age: 67 [SD: 9] years) with a range of glaucomatous visual field (VF) defects in both eyes (mean best eye mean deviation [MD]: –5.9 (SD: 5.4) dB) and 40 age-related people with normal vision (mean age: 66 [SD: 10] years) were timed as they searched for a series of target objects in computer displayed photographs of real world scenes. Eye movements were simultaneously recorded using an eye tracker. Average number of saccades per second, average saccade amplitude and average search duration across trials were recorded. These response variables were compared with measurements of VF and contrast sensitivity.

**Results:**

The average rate of saccades made by the patient group was significantly smaller than the number made by controls during the visual search task (P = 0.02; mean reduction of 5.6% (95% CI: 0.1 to 10.4%). There was no difference in average saccade amplitude between the patients and the controls (P = 0.09). Average number of saccades was weakly correlated with aspects of visual function, with patients with worse contrast sensitivity (PR logCS; Spearman’s rho: 0.42; P = 0.006) and more severe VF defects (best eye MD; Spearman’s rho: 0.34; P = 0.037) tending to make less eye movements during the task. Average detection time in the search task was associated with the average rate of saccades in the patient group (Spearman’s rho = −0.65; P < 0.001) but this was not apparent in the controls.

**Conclusions:**

The average rate of saccades made during visual search by this group of patients was fewer than those made by people with normal vision of a similar average age. There was wide variability in saccade rate in the patients but there was an association between an increase in this measure and better performance in the search task. Assessment of eye movements in individuals with glaucoma might provide insight into the functional deficits of the disease.

## Background

Patients with glaucoma commonly self-report problems with everyday vision-based tasks such as mobility, driving, reading and face recognition, which can have an adverse impact on their quality of life (QoL) [1-9]. Some attempts have been made to objectively characterise these difficulties in laboratory based experiments of ‘everyday’ vision-based tasks; for example, studies have reported that some patients with glaucoma display impairment in tasks such as reading [10], walking and balance tests [11-13], driving [14,15], reaching and grasping for household objects [16], face recognition [17] and visual search [18]. For the latter some patients with glaucomatous visual field (VF) defects in both eyes were shown to be significantly slower to locate target objects in computer displayed images of everyday scenes when compared to people with healthy vision of a similar age. Little is known about the underlying nature of the functional mechanisms influencing impairment from VF defects, but it seems likely that eye movements may play a role in this.

People make several eye movements per second, and where they look indicates what they perceive and see of the world. Saccades move the eyes in a ballistic fashion from one point to another, interspersed by periods of time where the eye is stable (fixations). Abnormal eye movement behaviour has previously been observed in patients with functional difficulties as a result of retinopathies other than glaucoma; for instance, evidence suggests a link between eye movements and impaired reading speed in age-related macular degeneration (AMD) [19,20]. Eye movement strategies have also been investigated during difficulties observed with mobility in retinitis pigmentosa (RP), with patients with increasing VF loss tending to fixate on alternative locations away from their intended goal when walking compared with controls with normal vision [21]. Moreover, training patients to alter the number of fixations they make has been shown to lead to improvements in their task performance [22,23] suggesting studies of eye movements in relation to ocular disease could potentially be useful for rehabilitation.

Remarkably little is known about the way in which patients with glaucoma move their eyes. Some evidence suggests that glaucomatous patients have more unstable fixations than age-matched control subjects, and that fixation stability is correlated with sensitivity in the central 10 degrees of VF [24]. However, this contrasts with earlier work showing that there was a large degree of variability in fixation accuracy across patients, but that this was unrelated to the extent of visual field loss at the test location [25]. A recent study examined eye movements made by a small group of glaucomatous patients as they searched for hazards in video clips of traffic scenes filmed from a driver’s perspective (The Hazard Perception Test [HPT]) [26]. On average, patients exhibited different eye movement characteristics to controls making, for example, significantly more saccades. This study aims to extrapolate this experimental paradigm by investigating eye movements in a larger group of patients as they interact with a ‘natural’ scene. In particular, the experiment described in this report is designed to test the hypothesis that patients with glaucomatous visual field defects in both eyes display different saccade behaviour when searching images of everyday scenes when compared to visually healthy individuals of a similar age.

## Methods

### Participants

Patients who had been previously diagnosed with Primary Open Angle Glaucoma, with repeatable visual field (VF) defects in both eyes were recruited from Moorfields Eye Hospital Trust London and the Fight for Sight Optometry Clinic at City University London. Patients had no ocular disease other than glaucoma. Control participants with healthy vision were selected from people attending Fight for Sight Optometry Clinic. Astigmatic error was less than ±2.5 Dioptres in all those recruited. Recruitment of patients and controls was made simultaneously with a specific effort to age-match participants. All original participants had reasonable general health (meaning no significant difficulty with self-care, mobility, pain, anxiety and depression). This was ascertained by self report to questions based on the EQ-5D instrument [27] added to the participation information sheet.

The study was approved by the Ethics Committee for the School of Health Sciences, City University London and the Moorfields and Whittington Local Research Ethics Committee. The study conformed to the Declaration of Helsinki and all subjects gave their informed written consent. All data was anonymised before being transferred to a secure computer database at the university.

### Vision testing, apparatus and procedure

Binocular visual acuity (measured using an Early Treatment Diabetic Retinopathy Study (ETDRS) chart) and contrast sensitivity (PR log CS) using a Pelli-Robson chart of all participants was measured prior to taking part in the study. To be included, participants were required to have a corrected visual acuity (VA) of at least 6/12 in each eye. Visual fields (central SITA 24–2 on both eyes) were also recorded on a Humphrey Visual Field Analyzer (HFA, Carl Zeiss Meditec, CA, USA) for all patients. Glaucoma Hemifield Test (GHT) was ‘outside normal limits’ for all patients and any VFs flagged by the HFA output as ‘unreliable’ (i.e. more than 20% fixation loss and more than 33% false positive and false negative error) were repeated until a ‘reliable’ VF was obtained. The HFA mean deviation (MD) is a standard clinical measure of the overall severity of a VF defect, with more negative values indicating greater VF loss and this was used as a measure of overall VF defect severity. Greyscales for integrated visual fields (IVF) were also constructed for each patient to give an estimate of binocular VF. This method involves the combination of the measured monocular VFs by simply taking the best sensitivity value at each point to represent the person’s binocular vision [28-30]. Absence of VF defects in the controls was confirmed by central SITA FAST 24–2 visual fields in both eyes. Participants were not recruited if they had any other ocular disease (except for an uncomplicated lens replacement cataract surgery). Patients had slit lamp biomicroscopy performed by an ophthalmologist to exclude ocular disease, especially any concomitant macular pathology. In order to further attempt to eliminate significant media opacity (cataract) and other lens type artefacts as confounding ocular conditions, all participants were required to be within ‘normal limits’ for abnormal light scattering in the eye media using the Oculus C-Quant straylight meter (Oculus GmbH, Wetzlar, Germany).

**Figure 1:**
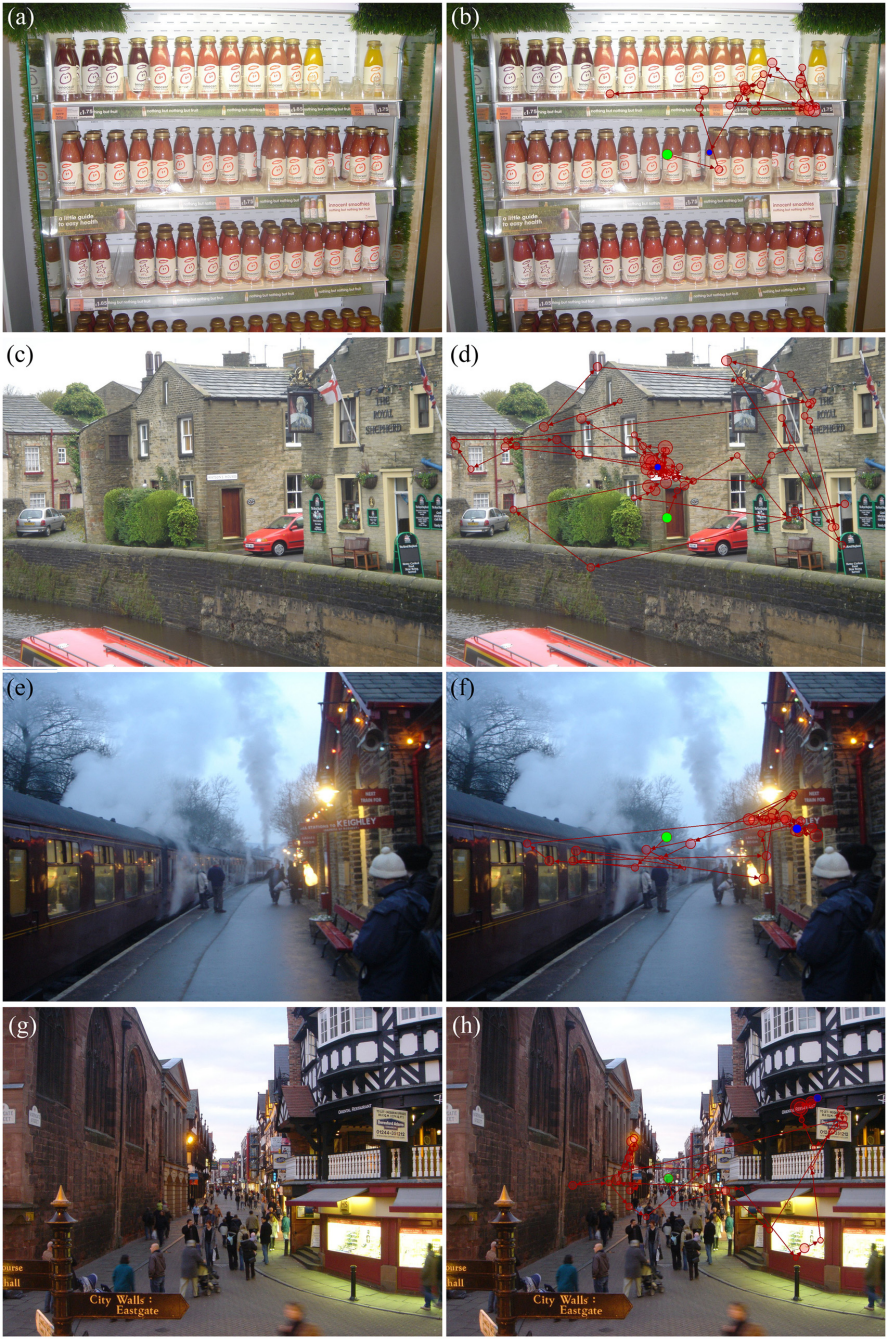
**Four different images from the study.** In image (**a**) participants were asked to find the price of the yellow smoothie drink. The task in image (**c**) was to find the name of the street. In image (**e**) participants were asked to find the place name for the destination of the train and in the final image (**g**) participants were asked to find the sign for the ‘Oriental Restaurant’. The same images are shown in (**b**), (**d**), (**f**) and (**h**) with superimposed scanpaths for four different patients. The green and blue symbols indicate the starting and final fixation respectively. The red circles represent fixations and connecting lines are saccades (circles increase in size with fixation duration).

The visual search task was performed on a 56 cm CRT computer monitor displaying at a resolution of 1600 × 1200 at a refresh rate of 100 Hz (Iiyama Vision Master PRO 514, Iiyama Corporation, Tokyo, Japan). Participants were presented with 15 digital photographs (and 3 practice images) of everyday scenes taken using the same camera (Sony DSC-T1, Sony Corporation, Tokyo, Japan), four of which are shown in Figure 1. These images were displayed in a darkened room at a resolution of 1600 × 1200 pixels and their mean luminance was 9.6 cd/m^2^ (SD: 4.0 cd/m^2^). Prior to the presentation of each image, the participant read a description of the target to be found on the screen; this instruction was read aloud simultaneously by the experimenter. Participants then fixated on a target point in the centre of the screen before the image was revealed, with the trial continuing until the person had informed the experimenter that they had found the target. All participants viewed the same 15 images but presented in a random order. When carrying out the task, the participants were positioned at a viewing distance of 60 cm and all the images displayed were 40.8 cm (width) × 30.6 cm (height) subtending a half-angle of 20.3° by 14.9°. All participants wore trial frames with a refractive correction suitable for the viewing distance of 60 cm to ensure that any obstruction to the field of view caused by spectacle frames would be equivalent for everyone.

During the visual search task, the participant’s eye movements were recorded using the Eyelink II system (SR Research Ltd., Ontario, Canada). The instrument was used to sample pupil position monocularly at 500 Hz (the chosen eye was alternated across participants). A chin rest was used to minimize head movements and patients were asked to keep their head as still as possible. Any head movements that did occur were compensated for by the EyeLink II’s head movement detection system which adjusts the point of regard accordingly. The EyeLink II proprietary algorithm was used to calibrate and verify the subject’s point of regard in relation to the correct location on the display. Calibration accuracy flagged by the system to be of a “good” level was a prerequisite before each trial. Therefore, before each image was displayed a drift correction was performed, and in the case where a large drift was detected, a recalibration performed. A participant’s verbal response that they had found the target was verified by the experimenter by ensuring that their point of regard, as measured by the Eyelink II, was superimposed on the target item. The trial was stopped at the moment the participant successfully located the target item and the time taken in seconds was recorded automatically by the eye tracking system. The main outcome measures of this experiment were the mean number of saccades made per second and the mean size of those saccades (saccade amplitude) across all trials. The median trial duration across all 15 images was also calculated to represent the participant’s average search time. All search times greater than 60 seconds were censored at this value.

### Analysis

The Eyelink II gives average eye position accuracy of better than 0.5° and uses velocity and acceleration thresholds of 30°/s and 8000°/s^2^ respectively to identify saccades. The application of these values is useful for filtering out ‘noisy’ eye-tracking data. For instance, the acceleration thresholds will reduce the likelihood that ‘noise’ will be classified falsely as a saccade. Furthermore, higher velocity thresholds will decrease the number of microsaccades that will be detected; whilst this information could potentially be useful in a purposely designed study, the focus of this work is primarily to analyse detected saccades in more complex images, and therefore the addition of microsaccades is likely to confuse the results. To remove potentially incorrectly classified saccades detected by the velocity and acceleration thresholds, all saccades with amplitudes less than 0.5° were excluded from the analysis. This saccade amplitude threshold was also applied in other studies that used the Eyelink to investigate eye movements when viewing images [31-33] and in this study, on average, 8.0% (SD: 0.03%) of the patient data and 8.1% (SD: 0.03%) of the control eye movement data was excluded. The average number of saccades per second and average saccade amplitude for each trial for each person was recorded. A General Linear Model (GLM) was used to perform a mixed two-way analysis of variance (ANOVA) to assess the eye movement parameters using SPSS 18 (IBM Corporation, Somers, NY, USA). Median imputation was used to compensate for very occasional instances whereby the eye tracker failed to record any data for a particular search trial. The two-way ANOVA arrangement was used to test the null hypothesis that variation in each response variable was not any different between the patients and controls examined (F test on the main factor, participant group, from the ANOVA). The GLM ANOVA is described as ‘mixed’ because the images are used as repeat measures allowing for an important assessment of interaction between performance and type of image, to verify that any differences are consistent across different images. Averages across the whole experiment (means for each eye movement parameter across all 15 test images separately) were also calculated for each participant and were plotted to illustrate overall effects, including overall variability within groups. For search duration, a replication of the analysis previously reported on a subsample of these patients was carried out [18]. Median search time across the 15 images was calculated. Search times are typically skewed so these averages were compared with a Mann–Whitney Test. Univariate associations (Spearman’s Rho) and stepwise multiple linear regression of eye movement parameters against age and severity of VF defect as measured by the Best Eye MD (MD of the better eye 24–2 HFA visual field) and severity of contrast sensitivity (PR log CS) was also performed in the patient group. Linear and 3 parameter exponential regression ( $y=a+b*e^{-\left(\frac{x}{c}\right)}$) using R (R Development Core Team, 2010) was used to examine the relationship between eye movements (saccades per second and saccade amplitude) and search performance (search duration).

## Results

Forty patients and 40 visually healthy age-related controls took part in the study. The patients and controls had a mean age of 68 (SD: 9) and 66 (SD: 10) years respectively. These means were not significantly different (two sample independent *t*-test; P = 0.49) and the spread of the distribution of ages were also similar (F-test of variances; P = 0.72) meaning the groups represent age-similar populations. All participants were of White Western-European origin. There were 20 (50%) men and 20 (50%) women in each of the patient and control groups respectively. The patients had a range of VF defect severity: average MD was −10.1 (SD: 7.5) dB, –8.2 (SD: 5.2) dB and −5.9 (SD: 5.4) dB in the right eye, left eye and best eye, respectively. Average Pelli-Robson contrast sensitivity values were significantly worse in the patients (mean: 1.83 [SD: 0.15] log units) compared to the control subjects (mean: 1.95 log units [SD: 0.01]) using a two-sample *t*-test (P < 0.001; 95% confidence interval [CI] for the mean difference of 0.08, 0.20). Mean ETDRS corrected binocular LogMAR VA was 0.04 (SD: 0.12) and −0.06 (SD: 0.10) in the patients and controls respectively. The difference between these mean values was statistically significant (P < 0.001; two-sample *t*-test) but the actual size of the average difference, 0.10, was small (95% CI for the mean difference of 0.05 to 0.16).

Median search time for the patients and the controls was 11.9 (inter quartile range [IQR]: 7.8 – 16.8) seconds and 8.1 (IQR: 5.9 - 10.2) seconds respectively: the difference in these median values was statistically significant (Mann–Whitney Test; P = 0.001) with a 95% CI for this difference ranging from 1.4 to 5.9 seconds. These results were similar to those previously reported in a smaller sample of people [18]. Consistent with this previous study, search durations were censored at 60 seconds. On average, 0.9 (SD: 0.81) trials per control and 1.4 (SD: 1.88) trials per patient were capped at 60 seconds before the item had been found. This difference was not statistically significant (two sample independent *t*-test: P = 0.13).

The eye movement parameters (saccades per second and saccade amplitude) were assessed separately using a mixed two-way GLM ANOVA with the images (trials) representing repeated measures. No obvious departure from Normality was observed in any of the average response variables as assessed by the Kolmogorov-Smirnov test. Where Mauchly’s test indicated that the assumption of sphericity did not hold, degrees of freedom were corrected using the Greenhouse-Geisser method. On average patients made significantly fewer saccades per second when compared to controls (F_1,78_ = 5.7; P = 0.02) and there was no significant interaction term (F_9,720_ = 1.1; P = 0.37; groups by image) meaning that the effect was consistent across all the 15 repeat measures (trials). There was, however, no real statistical evidence for a difference in the mean saccade amplitude between the groups (F_1,78_ = 2.9; P = 0.09).

**Figure 2:**
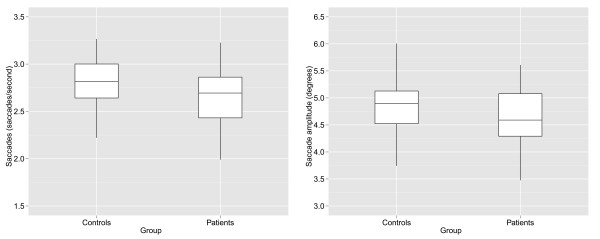
Box plots showing mean differences between controls and patients for (a) saccades per second and (b) saccade amplitude.

Figure 2 (a, b) illustrates the results from the search task by plotting overall mean values for the eye movement summary measures for each participant in the experiment. The overall mean number of saccades per second was 2.65 (SD: 0.3) for the patients and 2.81 (SD: 0.3) for controls, equating to an average reduction in saccade numbers of 5.6% (95% CI: 0.1 to 10.4%). The overall mean saccade amplitude (in degrees) was 4.6 (SD: 0.5) and 4.8 (SD: 0.5) for patients and controls.

**Figure 3:**
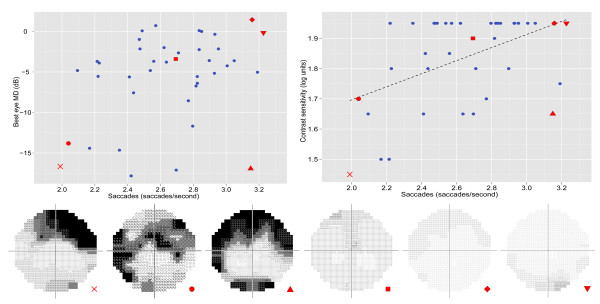
**Scatterplots showing the link between saccade rate and BEMD and PR log CS respectively in the n = 40 patients.** Linear regression was performed between the saccade rate and PR log CS, and is represented by the black line. The integrated visual fields (IVF) are shown for 6 patients at different saccade rates. Scanpaths for two of the patients, represented by the cross and square red symbols, were shown in Figure 1 (images b and d respectively). Dynamic scanpaths of these patients are also highlighted in the videos in the additional files.

In the patient group (n = 40) there was a weak but statistically significant univariate association between number of saccades per second and best eye MD (rho = 0.34; P = 0.037). This association was slightly stronger between number of saccades and PR log CS (rho = 0.42; P = 0.006) (Figure 3). In both cases the regression only accounted for less than approximately 20% of the variance in these data. There was no statistically significant association between saccade amplitude and the visual function measures (Best eye MD rho = 0.20, P = 0.22; PR log CS rho = −0.02, P = 0.91).

A stepwise linear regression was performed (criteria: probability of F to enter ≤ 0.05, probability of F to remove ≥ 0.1) in the patient group (n = 40), using saccade rate as the dependant variable, age as a fixed predictor to ensure the effects of the other variables can be determined independently, and CS and best eye MD (BEMD) as the predictors. CS was included in the final model (P = 0.03; R^2^ = 27.4%) and BEMD (P = 0.61) excluded. None of the variables met the stepwise criteria for saccade amplitude.

**Figure 4:**
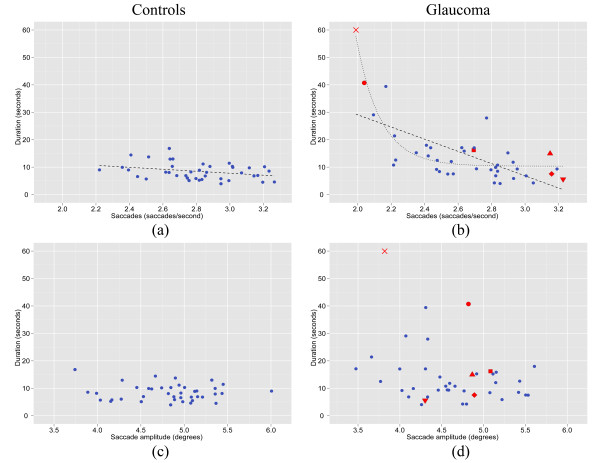
**Duration to find the target within the image compared to saccades per second (a, b) and saccade amplitude (c, d) in the real-world search task for the n = 40 controls and n = 40 patients respectively.** Univariate linear regression was performed for saccade rate compared to search duration in both the control (**a**) and patient (**b**) groups. Further to this, exponential regression (a 3 parameter exponential regression $y=a+b*e^{-\left(\frac{x}{c}\right)}$ where x is represented by saccade rate) was applied to the latter, which is approximately 8 million times more probable when comparing its AIC to that of the linear model (**b**) [48]. The red coloured symbols within the patient plots correspond to those patients highlighted in Figure 3. Note, for example, that patients represented by the red circle and triangle symbols have similar levels of VF defect severity (see Figure 3), but very different search duration performance.

When investigating the relationship between search performance (search duration) and the number of saccades made per second in the patient group, Spearman’s correlation coefficient revealed a statistically significant association (rho = −0.65; P = 0.001). There was no relationship between saccade amplitude and search performance (rho = −0.20; P = 0.21). Figure 4 shows the scatterplots relating search performance to the eye movement parameters. These scatterplots indicate that the relationship might not be a linear one (in the patients) with some evidence that search performance rapidly declines as an exponential function as the saccade rate declines [48]. For example, whereas there was a large increase in search duration as saccade rates were less than roughly 2.1 saccades per second, above this rate there was very little change in duration as a function of increasing saccades per second. This observation is supported by the R^2^ associated with the exponential fit (R^2^: 76%) as compared to the linear fit (R^2^: 43%).

### Videos of dynamic scanpaths

Additional file 1 contains a video displaying the eye movements of one patient (IVF in Figure 3 represented by the blue coloured symbol) and the eye movements of a sample of controls. In this video the blue symbol indicates the point of regard for the patient and the red symbols represent the point of regard for a random sample of the controls. When a symbol disappears it means that the particular person found the target and completed the task (in this case, finding the price of the yellow coloured drink). Additional file 2 shows the same sequence but with the patient’s IVF superimposed and moving with fixation; the less transparent areas equate to defects in the IVF. In this case, the location of the superior binocular VF defect initially masks the target and this might explain the slow search time. (Note: The superimposed IVF does not represent how a glaucomatous patient sees, but gives an indication of why they might struggle with the task). Similarly Additional files 3 and 4 show dynamic eye movements and search performance for another patient (IVF in Figure 3 represented by the brown coloured symbol) on another image where the task was to identify a street sign. This example shows the difficulty this patient has with the task because of their central binocular defect.

## Discussion

Searching for objects is an important everyday task; for instance, looking for an item on a supermarket shelf, a sign in a railway station or a place name on a map will require the swift detection of the ‘target’ object amongst an array of irrelevant ‘distractor’ items. The results from this study confirm previous findings about how VF defects can negatively impact on the success of visual search [18]. This study adds to this knowledge by demonstrating that the saccadic behaviour of patients may underpin these deficits, with a strong association, in patients, between time to find targets and saccade rate. Moreover, the average number of saccades per second for the patient group in this study was significantly smaller than the average in the visually healthy group of a similar age. This report also provides novel information because eye movement studies in large groups of elderly people are uncommon.

Peripheral vision plays a key role in saccadic eye movement behaviour, in that it is necessary for detecting the objects of most functional importance so that a subsequent saccade can be made towards it, bringing the item onto the fovea for more detailed inspection [34-36]. One explanation for the patient group exhibiting fewer saccades on average when compared to the controls could be the degradation of peripheral vision, meaning the patient struggles to detect the most salient items around them and, subsequently, struggles to initiate saccades towards these regions. Similarly, changes in eye movement behaviour (reduction in saccade initiation) has also been observed when the VF is obstructed with an artificial scotoma in observers with normal vision [37]. Likewise, it has been shown that manipulating the size of high-resolution area on an image available to observers with normal vision also directly affects eye movements when searching for items within the images; a greater region of high-resolution information was associated with more saccades and more efficient visual search [38]. This offers an explanation for the results in the patients because increasingly poorer visual quality in the periphery will likely decrease the number of saccades that can be made, and subsequently affect search performance.

This study indicated that average saccade amplitude in the patient group did not differ from the average in the visually healthy individuals. This finding contradicts previous research suggesting that people make saccades of smaller amplitudes when their vision is degraded in order to avoid the affected areas [37,38]. These differences could be related to the fact that using artificial targets in a controlled experiment is very different from searching for features in ‘natural’ scenes. Moreover, the effects of manipulating the characteristics of an artificial scotoma in participants with normal vision will likely not be equivalent to the experiences of patients with glaucomatous VF defects. Nevertheless, it would be reasonable to expect patients to make smaller saccades within intact regions of vision and so the lack of significant effect found here is surprising. The results may simply reflect the large variability in defect size and location seen within the patients in this study; future studies exerting better control over defect type might therefore yield more information regarding the specific effects of VF loss on saccade amplitude.

There was large between-patient variability for number of saccades made. Perhaps some patients have adapted to make more eye movements than others, and this in turn affects search performance (See, for example, patients highlighted in Figures 3 and 4). Of course, visual characteristics of the patients might help determine what may be driving these differences. Contrast sensitivity was found to be significantly associated with the number of saccades made during the search task. CS loss often coincides with glaucomatous damage [39,40] and so its influence on the eye movement strategies used by the patients is somewhat expected. Stimulus contrast is an important visual property for dictating eye movement behaviour, with evidence suggesting that stimuli with higher contrast are more likely to be selected for fixation [41]. A patient with diminished contrast sensitivity might subsequently be less likely to detect such key items and will thus likely require more time to build up a detailed representation of the visual scene. There was also a significant association between the number of saccades and best eye MD, suggesting that fewer saccades in glaucoma is in some way linked to the severity of the VF defect. All these associations are relatively weak but this could be attributed to the ‘bluntness’ of the clinical measures used for visual function. The videos in the additional files showing individual cases illustrate how eye movements should be correlated with the location of the VF defect, but quantification of this awaits further study.

This study also found that the 40 patients took longer, on average, to find the target objects than the visually healthy controls, thereby confirming previously published findings based on a smaller sample of participants [18]. However, it is important to note that not all patients displayed poorer search times than the controls, and that visual search disability is therefore not a certainty in patients with glaucoma. This variability in task performance has also been observed in other studies assessing disability in glaucoma using performance-based measures [16,17]. The average drop in saccade rate observed in the patient group may therefore help explain this apparent functional deficit. Likewise, there was some variability in terms of the number of saccades made by the patients during the current task. Interestingly, analyses between eye movements and search times revealed that the eye movements produced by the patients were linked to search times: the more saccades made by a patient per second, the less time it took them to find the target. Estimating the exact nature of this relationship is beyond this single experiment but there was some evidence of a linear association but more for an exponential relationship. Under the assumption of the latter, it appears that those patients with very low saccade rates are particularly impaired in search performance. The sample of patients tested in this study was quite large but it might be more fruitful to deliberately identify, or recruit patients with saccade rates or search times that are exceptionally diminished for further study on other tasks. In that regard, much can be learnt from observing the single patients highlighted in the movies shown in additional files***.*** The relationship between number of saccades and performance was less apparent in the controls suggesting that the eye movement differences observed might be the result of some sort of adaptive strategy that may be induced as a result of glaucomatous VF defects in some patients. In turn this could suggest that the eye movement strategies used by patients with glaucoma may influence the likelihood of them experiencing functional task deficits, providing an additional explanation for the individual variability observed in search times amongst patients in our previously reported visual search work, and in studies involving other visual tasks [10,16,18]. Training patients with AMD to make increasingly large eye movements in reading tasks has been shown to lead to improvements in reading speed [22]. Also, training individuals with hemianopia to make larger saccades in their blind regions has been shown to increase the field of view for visual search and subsequently improve performance [42,43]. It is not unreasonable to suggest that similar rehabilitative approaches might benefit some patients with glaucoma, especially encouraging making more saccades during search, but this speculation awaits better characterisation of the specific types of defects that are more or less associated with task restriction and manifested eye movements.

Some of the results reported in this study were orthogonal to those reported before; for example, we previously reported an *increase* in saccade rate when patients viewed moving driving scenes (Hazard Perception Test; HPT) [26]. This was, however, a different task with a dynamic image rather than a still one. Generally, it is accepted that individuals will alter their eye movements in accordance with how difficult a task is, with more difficult tasks leading to the use of more fixations and saccades to process the scene [44,45] and this is reviewed in detail elsewhere [46]. The dynamic, fast-paced HPT likely requires more cognitive effort, and thus more saccades, to sufficiently process the scene when compared to the still images presented in this study. These differences reinforce the importance of considering a variety of visual tasks to gain an insight into the nature of eye movement changes that may be caused by glaucoma.

Some limitations of this study should be highlighted. For example the visual search task had a small number of trials (n = 15), designed to eliminate fatigue effects, but this makes our estimates less reliable than if more trials were employed. The images were two-dimensional and static, and therefore not exactly reflective of the dynamic and changeable nature of the real world. Furthermore, other factors may have influenced our search duration recordings; for instance, whilst the eye tracker recorded whether a person’s gaze corresponded with the item to be found, a trial was only stopped once a person had verbally confirmed that they had found the target. Therefore, how certain a person felt before they made their decision will have influenced their overall search time. It may be that higher levels of uncertainty about a target corresponded to certain properties of the image, such as target areas with low levels of luminance or contrast; however there was insufficient information to be able to quantify this in the current study. The experimental design also means that it is unclear at this stage what exactly may be driving the apparent differences in eye movement behaviour. The patients and controls were age similar but we did not consider other confounders such as cognitive ability or reduced colour vision; deficits in the latter can be a feature of glaucoma [47]. It is possible that some of the study’s inclusion and exclusion criteria, whilst serving to exert control over factors such as the ocular disease measured (*only* glaucoma), and including those in good general health (other than glaucoma), may have introduced additional bias into the results.

## Conclusion

The average number of saccades made per second when searching for objects in everyday images by this group of patients with glaucomatous VF defects in both eyes was fewer than those made by people with normal vision of a similar average age. The size of the average difference was relatively small and there was wide variability in the measurements within the patient group. Put differently, not all patients would have eye movement behaviour that would fall outside the range of those exhibited by people with normal vision. However, the saccadic behaviour of the patients was related to aspects of their visual function; for instance, there was an association revealing patients with more severe VF defects (worsening MD in their best eye) and worsening contrast sensitivity manifested fewer saccades. Furthermore, this study adds new knowledge by demonstrating that saccade rate made by the patients, but not the controls, is associated with the search time; inferring that eye movement behaviour underpins some of the problems with visual search observed for certain patients. In turn this may suggest that an increased saccade rate could allow some patients to be more efficient in the task of searching. It would be interesting to investigate if this is a form of adaptation, consciously performed or otherwise, to glaucomatous visual loss. Indeed, these results are reported to stimulate further study of eye movements during everyday tasks to increase understanding of visual functioning in glaucoma.

## Competing interests

The authors declare that they have no competing interest.

## Authors' contributions

NDS participated in the design of the study, collection and analysis of the data and writing of the manuscript. FCG assisted with data collection and the writing of the manuscript. DPC conceived and designed the study and revised the manuscript.

## Pre-publication history

The pre-publication history for this paper can be accessed here:

http://www.biomedcentral.com/1471-2415/12/45/prepub

## Supplementary Material

Additional file 1 **Video 1.** Video showing the fixations made by a patient (blue point) and the controls (red points) when searching for the price of the yellow drink (located at the top right of the image). Data corresponding to the patient can be located in Figures 3 and 4 by thecross symbol. Note that once the point disappears the person has located the object and their trial has ended. Click here for file

Additional file 2 **Video 2.** Video showing the fixations and partial scanpath of a patient searching for the price of the yellow drink (located at the top right of the image). The patient is the same as in Additional file 1. The overlaid semi-transparent sections relate to the patient’s IVF sensitivity values: the less transparent the region is, the lower the patient’s sensitivity at that location of their IVF. Click here for file

Additional file 3 **Video 3.** Video showing the fixations made by a patient (blue point) and the controls (red points) when searching for the street sign (located at the centre of the image). Data for the patient is represented in Figures 3 and 4 by the square symbol. Note that once the point disappears the person has located the object and their trial has ended. Click here for file

Additional file 4 **Video 4.** Video showing the fixations and partial scanpath of a patient searching for the street sign (located at the centre of the image). The patient is the same as in Additional file 3 and the visual field representation is in the same format as Additional file 2. Click here for file
